# Ikaros and *RAG-2*-Mediated Antisense Transcription Are Responsible for Lymphocyte-Specific Inactivation of *NWC* Promoter

**DOI:** 10.1371/journal.pone.0106927

**Published:** 2014-09-08

**Authors:** Agnieszka Łaszkiewicz, Łukasz Bzdzion, Monika Kasztura, Łukasz Śnieżewski, Sylwia Janik, Paweł Kisielow, Małgorzata Cebrat

**Affiliations:** Laboratory of Molecular and Cellular Immunology, Department of Tumor Immunology, Institute of Immunology and Experimental Therapy, Wrocław, Poland; Chang Gung University, Taiwan

## Abstract

Recombination activating gene-2 (*RAG-2*) and *NWC* are strongly evolutionarily conserved overlapping genes which are convergently transcribed. In non-lymphoid cells the *NWC* promoter is active whereas in lymphocytes it is inactive due to the DNA methylation. Analysing the mechanism responsible for lymphocyte-specific methylation and inactivation of *NWC* promoter we found that Ikaros, a lymphocyte-specific transcription factor, acts as a repressor of *NWC* promoter - thus identifying a new Ikaros target - but is insufficient for inducing its methylation which depends on the antisense transcription driven by *RAG-2* promoter. Possible implications of these observations for understanding evolutionary mechanisms leading to lymphocyte specific expression of *RAG* genes are discussed.

## Introduction

**Figure 1:**
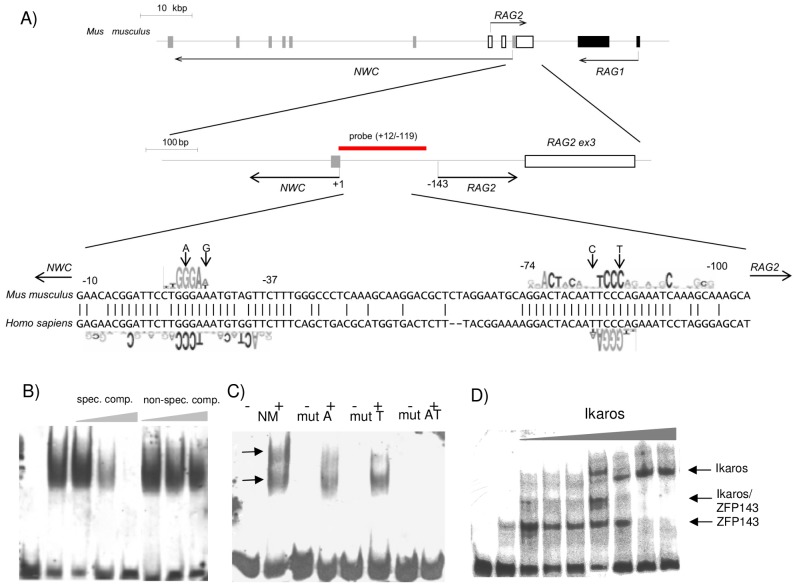
Ikaros-binding properties of *NWC* promoter. (A) Structure of *RAG/NWC* locus and *NWC* gene promoter. Open gray and black boxes represent *NWC*, *RAG-2* and *RAG-1* exons, respectively. Horizontal arrows indicate the directions of the transcription and the numbers indicate the position of the sequences relative to *NWC* transcriptional start site. Red line indicates the localization of the EMSA probe (−119/+12) lying within the *NWC* promoter (−119/+125). Aligned sequences of murine (*Mus musculus*) and human (*Homo sapiens*) *NWC* promoters are shown. The sequence logos represent ZFP-143 binding sites and putative Ikaros binding sites. Vertical arrows indicate the location and nature of the mutations introduced in probes and reporter constructs used throughout this study. (B) EMSA experiment showing Ikaros binding to *NWC* promoter: first lane from the left – free probe, second – probe and Ikaros, next – probe, Ikaros and increasing molar excess (10, 50, 100x) of unlabelled specific (lanes 3–5) or non-specific competitors (Oct-2, for sequence see Materials and Methods) (lanes 6–9). (C) EMSA experiment showing Ikaros binding to non-mutated (NM), single mutated (mutA, mutT) or double mutated (mutAT) NWC promoter. Probes were tested in the absence (−) and presence (+) of Ikaros. (D) Competition of ZFP-143 and Ikaros for *NWC* promoter binding. Constant amount (150 ng) of ZFP-143 protein and increasing amount of Ikaros protein (Ikaros/ZFP-143 molar ratio: 0, 0.3, 0.5, 1, 1.5, 2, 2.5, 3, lanes 2–8) were used to bind to the probe corresponding to non-mutated *NWC* promoter.

*NWC* (“Nad Wyraz Ciekawy”, which translates from Polish to “extremely interesting”) is the third evolutionarily conserved gene within the recombination-activating genes *RAG-1* and *RAG-2* locus [1] encoding a protein complex indispensable for the recombination of immunoglobulin and T-cell receptor minigenes [2]–[5]. The popular hypothesis on the origin of *RAGs* proposes that the transposon containing one of or both *RAG* genes infected a germ cell of an ancestor of jawed vertebrates (or deuterostomes), ultimately allowing for the development of lymphocytes [6], [7]. The first exon of *NWC* gene and its promoter are located in the intron preceding the coding exon *RAG-2* gene and these two genes are convergently transcribed [1] (Fig. 1A). The promoter of *NWC* gene is active in non-lymphoid cells and exhibits bidirectional activity, which can drive the transcription of both *NWC* and *RAG-2* transcripts in some non-lymphoid cells [8]. Based on this observation, we have recently proposed that the bidirectional activity of *NWC* promoter could facilitate the integration and survival of *RAG* transposon in the ancestral genome [8]. Previously, we suggested that *NWC* transcription may negatively control *RAG-1* and *RAG-2* promoter activities in non-lymphoid cells owing to transcriptional interference caused by *NWC* transcription proceeding through *RAG-2* promoter and *RAG-1/RAG-2 cis*-regulatory elements localized upstream *RAG-2* gene [9]. This hypothesis has not been verified so far, since due to the remaining activity of a secondary promoter [10], we have been unable to abrogate completely the transcription of *NWC* in mice, in which primary *NWC* promoter was deleted.

The primary *NWC* promoter is associated with a CpG island which is unmethylated in non-lymphoid cells and becomes methylated in immature T- and B- lymphocytes, which coincides with the promoter's inactivation [11]. In lymphocytes the function of *NWC* promoter is taken over by *RAG-1* promoter, which results in the expression of *RAG-1/NWC* hybrid transcripts [1]. The methylation of *NWC* promoter is not accompanied by other changes in chromatin organization, i.e. changes in posttranslational modifications of histone H3 [11] that are commonly associated with transitions between transcription permissive and repressive chromatin configuration and usually precede DNA methylation. Blocking DNA methylation with 5-azacytidine partially restores the activity of *NWC* promoter in lymphocytes [11], proving the primary role of DNA methylation in controlling its activity. The activation of *NWC* promoter is mediated by ZFP-143 transcription factor which binds to its two conserved elements, also possessing consensus binding sites for Ikaros transcription factor [8].

Ikaros is an essential transcription factor required for lympocyte development. It is expressed in lymphoid cells, haematopoietic stem cells and some myeloid cells. Ikaros deficiency impairs the development of lymphoid and myeloid cell lineages [12]. Ikaros can be involved both in gene activation and repression and its activity occurs at different levels: by direct competition with the activator proteins for common binding sites at the target promoter [13], by restructuring chromatin through targeting different types of chromatin remodelling factors [14]–[16] such as SWI/SNF (activator) or NuRD deacetylase (repressor) as well as by bridging the target genes destined for inactivation with centromeric foci, thereby facilitating their assembly into pericentromeric heterochromatin [17]. Ikaros target genes include *RAG-1* and *RAG-2* genes, which are tightly controlled throughout lymphocyte development. High and coordinated expression of *RAG* genes is regulated by the activity of several *cis*-elements localized mainly upstream *RAG-2* gene [18]–[21]. Investigating the role of Ikaros in regulating V(D)J recombination in B-cell lineage Reynaud and collegues [22] showed that Ikaros binds directly to regulatory elements of *RAG* locus in pro-B cells, namely with the Ep, D3, E-rag enhancers and *RAG-1* promoter but not to *RAG-2* promoter. These authors also compared the histone modification status of regulatory elements in *RAG2* ^−/−^ vs Ikfz^−/−^ pro-B cells and concluded that activation of *RAG* transcription by Ikaros is accompanied by histone-H3 acetylation. Here we demonstrate that *NWC* represents a new target of Ikaros activity within *RAG* locus. We show that binding of Ikaros to *NWC* promoter downregulates *NWC* expression, but is unable to cause promoter methylation which is established by antisense transcription driven by the activity of *RAG-2* promoter. We discuss how these two mechanisms: binding of Ikaros and antisense transcription may act in concert to inactivate *NWC* promoter.

## Results

### Ikaros binds to *NWC* promoter

We have recently shown that the promoter of *NWC* gene is activated by ZFP-143 transcription factor, which binds two inverted evolutionarily conserved sites of the promoter [8] spanning −10/−37 and −74/−100 nucleotides relative to transcriptional start site. We noticed that these regions also contain consensus binding sites for Ikaros transcription factor (TGGGAA) [12], which overlap with the ZFP-143 binding sequences (Fig. 1A). This observation raised the possibility that Ikaros could inactivate *NWC* promoter by competing with ZFP-143. As a first step towards verifying this possibility we checked whether Ikaros is able to bind to the promoter using electromobility shift assay (EMSA). The recombinant HisTag-Ikaros protein yielded two complexes with the probe corresponding to −119/+12 portion of *NWC* promoter (Fig. 1B). The slow-mobility complex was disrupted when the probes mutated at one of the two binding sites (TGAGAA) were used, while mutation of both binding sites resulted in complete disappearance of slow- and fast-migrating complexes (Fig. 1C). These results suggested that Ikaros is able to bind simultaneously and independently to both predicted sites in the promoter, which indicates that ZFP-143 and Ikaros share the same binding sites containing four strict consensus nucleotides (TCCC) indispensable for binding both proteins. In order to confirm this conclusion we compared their binding to the promoter in an EMSA competition experiment. Addition of increasing concentrations of HisTag–Ikaros to binding reactions containing the *NWC* promoter probe and a constant amount of ZFP-143 led to a gradual reduction in the abundance of the ZFP-143-containing complex. Addition of Ikaros in a ratio ranging from 0.3∶1 to 2∶1 produced an intermediate migrating complex containing presumably one molecule of Ikaros and ZFP-143, whereas addition of Ikaros in a ratio of 2.5∶1 resulted in the disappearance of fast (ZFP-143) and intermediate (ZFP-143/Ikaros) complexes, producing ones containing only Ikaros (Fig 1D).

### Ikaros downregulates the expression of *NWC*

**Figure 2:**
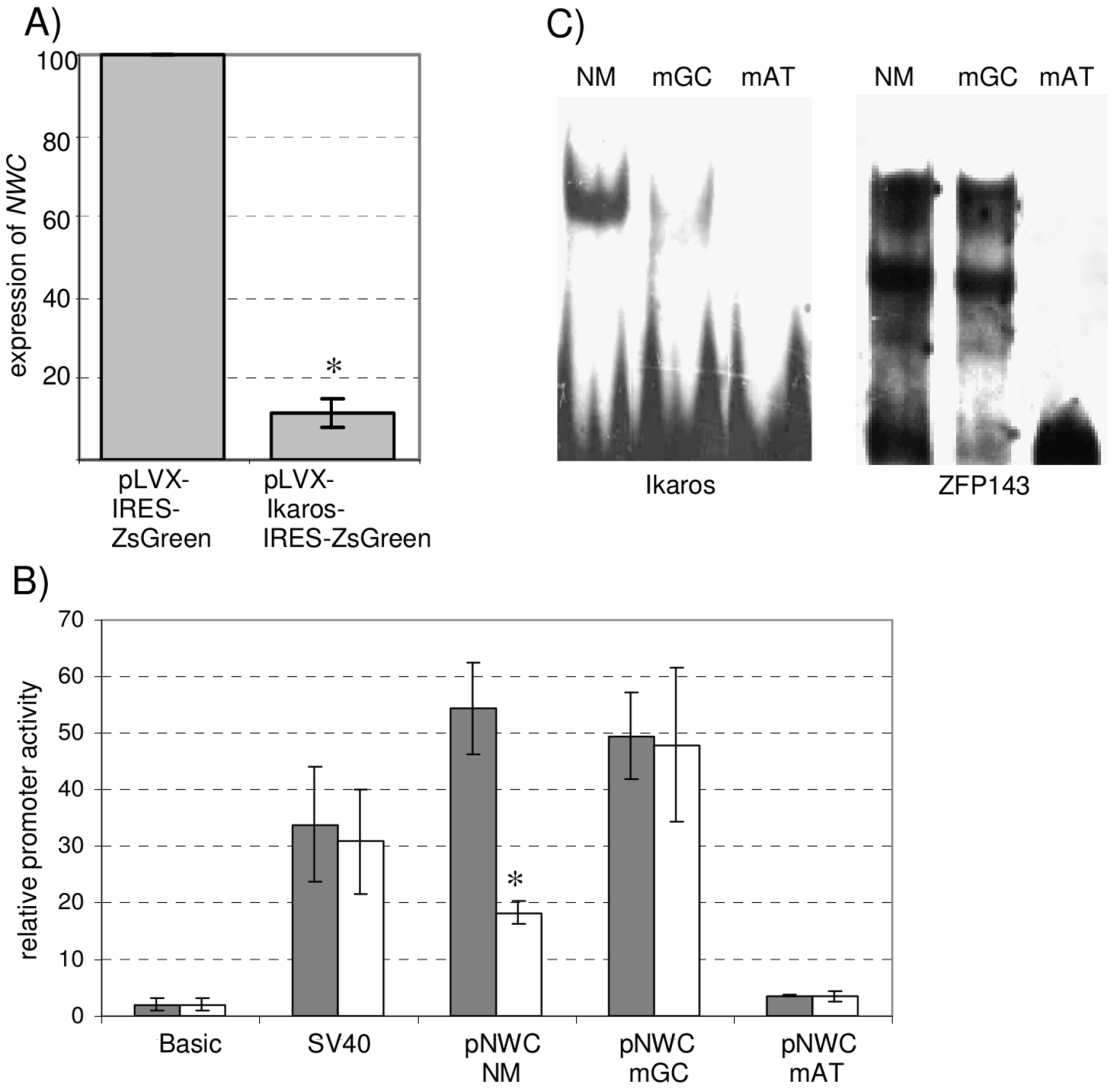
Ikaros downregulates the activity of *NWC* promoter. (A) Real-Time RT-PCR analysis of endogenous *NWC* expression in non-lymphoid cells (HEK293T) transfected with Ikaros-expressing construct (pLVX-Ikaros-IRES-ZsGreen) or with empty vector (pLVX-IRES-ZsGreen) (100%). Transfection efficiency varied and approximated 80%. The MFI after sorting was ∼1300. Expression values were normalized to *GAPDH*. The results shown are the means of three experiments with error bars representing ±1 SD. The asterisk indicates a significant difference (p<0.05) between both sample groups. (B) Activity of *NWC* promoter fragments containing point mutations in the Ikaros/ZFP-143 binding sites tested by luciferase assay in HEK293T cells ectopically expressing Ikaros gene (white bars) or transfected with empty vector (grey bars). pNWC-NM: non-mutated binding sites, pNWC-mGC: mutation specifically affecting the Ikaros binding site (see C), pNWC-mAT: mutation affecting both Ikaros and ZFP-143 binding sites. The relative promoter activities were normalized to the activity of promoter-less vector (pGL3-Basic). Vector containing SV40 promoter was used as a control. The results shown are the means of three experiments with error bars representing ±1 SD. The asterisk indicates a significant difference (p<0.05) between the activity of the given promoter in the presence or absence of Ikaros expression. (C) EMSA experiments verifying the Ikaros-specific nature of mutations (mGC) introduced to *NWC* promoter constructs. Non-mutated probes (NM) and probes with mutations affecting both Ikaros and ZFP-143 binding (mAT) were used as controls to monitor the efficiency of Ikaros and ZFP-143 binding.

In order to determine whether Ikaros influences the expression of *NWC*, HEK293T cells were transfected with pLVX Ikaros-IRES-GFP expression vector. Transfected cells were sorted based on the high GFP expression and assayed for the expression of *NWC* in Real-Time RT-PCR assay. As shown in Fig. 2A, overexpression of Ikaros led to a significant (∼7 times) downregulation of *NWC* expression. In order to find out if the downregulation is due to a direct interaction of Ikaros with *NWC* promoter we tested the effect of Ikaros overexpression in cells co-transfected with reporter vectors containing *NWC* promoter constructs. Figure 2B shows that the overexpression of Ikaros resulted in a significant reduction of the promoter activity of constructs containing an *NWC* promoter fragment (−119/+125 relative to the *NWC* transcription start site) but had no effect on the control SV40 promoter. In order to confirm the specificity of this effect we used *NWC* promoter fragments containing point mutations affecting the consensus binding sequences for both Ikaros and ZFP-143 (mAT) or for Ikaros alone (mGC). The influence of introduced mutations on the ability of Ikaros and ZFP-143 to bind the constructs was verified by EMSA (Fig. 2C), confirming Ikaros-specific nature of mGC mutation. As expected, promoter containing mutations in binding sequences for both ZFP-143 and Ikaros showed significantly reduced activity when compared to a non-mutated promoter and the effect was not further enhanced with the overexpression of Ikaros. The promoter fragment containing the mutations in Ikaros binding sequences had similar activity as the non-mutated promoter, but, in contrast to the latter, its activity was not reduced after the overexpression of Ikaros (Fig. 2B). In order to determine whether the reduction in *NWC* promoter activity caused by Ikaros overexpression is accompanied by the methylation of *NWC* promoter we performed bisulfite sequencing of *NWC* promoter using HEK293T cells transfected with pLVX Ikaros-IRES-GFP vector. Ikaros overexpression did not result in any changes in the methylation status of *NWC* promoter as compared to non-transfected cells: *NWC* promoter was unmethylated in both cell types (0%–1%) (not shown). Lymphoid cell line (Jurkat) was used as a control in this experiment and was shown to have completely methylated *NWC* promoter. Altogether these results indicate that Ikaros is able to bind to *NWC* promoter and reduce its activity owing to the competition with ZFP-143 transcriptional activator for common binding sites but alone is not sufficient for the methylation of *NWC* promoter.

### *RAG-2* antisense transcription is responsible for *NWC* promoter methylation

**Figure 3:**
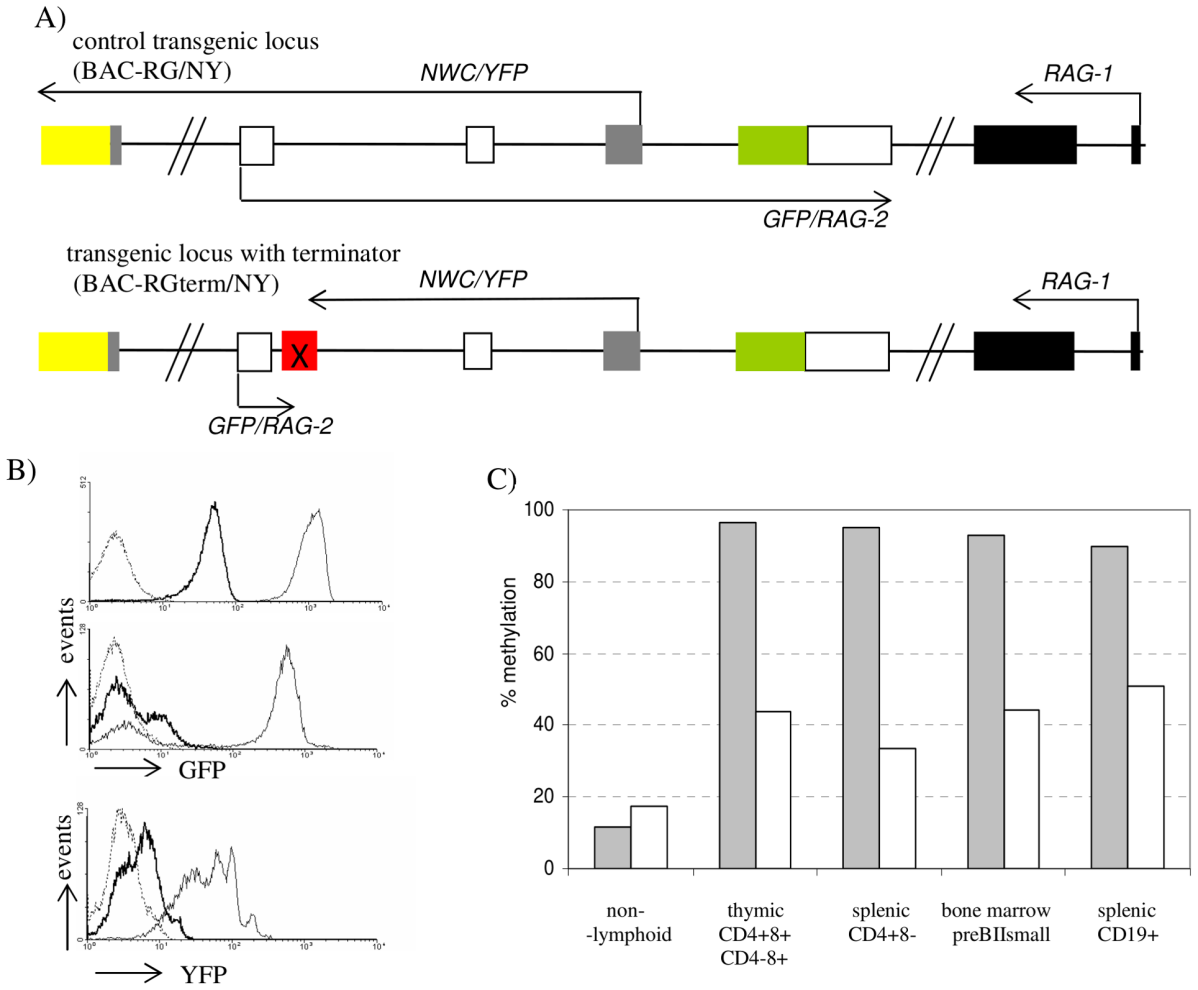
*RAG-2* transcription is responsible for *NWC* promoter methylation. (A) The *RAG/NWC* locus of BAC-RG/NY and BAC-RGterm/NY transgenic mice. Relative positions of the exons encoding *RAG-1* (black boxes), *RAG-2* (open boxes), and *NWC* (gray boxes) are shown. Horizontal arrows indicate transcription start sites and orientations. Modifications of the locus are presented as green, yellow and red boxes representing *GFP*, *YFP* and the transcriptional termination cassette, respectively. (B) Cytofluorimetric evaluation of RAG-2/GFP protein expression in double positive thymocytes (upper histogram) and bone marrow B lymphocytes (middle histogram) and NWC-YFP in testis (lower histogram) of BAC-RGterm/NY (bold line), BAC-RG/NY (normal line) and non-transgenic control (dashed line). (C) Methylation status of transgenic *NWC* promoter of control (grey bars) and *RAG-2* terminator-containing (white bars) loci.

Searching for a mechanism that could be responsible for lymphoid-specific methylation of *NWC* promoter we focused our attention on *RAG-2* transcription, which, because of a convergent transcription of these two genes, represents an antisense transcription in relation to *NWC*. In order to test if *RAG-2* transcription influences the methylation status of *NWC* promoter we have generated a transgenic mouse strain using BAC-based transgene containing complete murine *RAG/NWC* locus modified to express *GFP* under the control of *RAG-2* promoter [18]. We have modified this transgene by inserting *YFP* gene in frame with *NWC* gene and a transcriptional termination cassette consisting of two SV40-polyA sequences linked with twelve *lacO* operators [23] immediately downstream of *RAG-2* first exon (BAC-RGterm/NY). A transgene without the termination cassette was used to generate the control mouse strain (BAC-RG/NY) (Fig 3A). The offspring of the transgenic founder animals was analyzed by flow cytometry. As expected, in the BAC-RG/NY control mouse, the highest level of the expression of *RAG-2/GFP* was detected in developing thymocytes and B lymphocytes while the highest expression of *NWC/YFP* was detected in testis (Fig. 3B) consistent with our previous findings [1], [10] that these cells express the highest level of *NWC* transcript. Figure 3B also shows that the BAC-RGterm/NY mice showed a strong reduction of expression of *RAG-2/GFP* providing evidence that the transcriptional termination cassette was functional. Since the cassette was also found to be bidirectional, it strongly reduced the expression of NWC-YFP reporter (Fig 3B) and made it impossible to monitor the potential effect of *RAG-2* transcription termination on *NWC* promoter activity by flow cytometry. Therefore, we analyzed the methylation level of *NWC* promoter by bisulfite sequencing. Using transgene-specific primers (i.e. one of the primers in each pair in the nested PCR was complementary to *GFP*) we were able to distinguish transgenic from endogenous loci and thus determine the influence of the termination of *RAG-2* transcription on *NWC* promoter methylation. As shown in Figure 3C, the termination of *RAG-2* transcription significantly reduced the methylation level of *NWC* promoter as compared to the control mice, indicating that the methylation is due to a *cis*-mechanism. In double positive (CD4+8+) thymocytes, single positive thymocytes, bone marrow preBII small cells and splenic B cells the methylation level was reduced from nearly 100% to ∼40%. Importantly, in non-lymphoid tissues (liver, brain and testis) of both mouse strains, the methylation level of *NWC* promoter was similarly low (12%–17%), indicating that the differences in promoter methylation observed in lymphocytes were not due to the position effect of the integrated transgene.

## Discussion

In the present study we have shown that two mechanisms contribute to the lymphocyte specific inactivation of *NWC* promoter: Ikaros induced repression and DNA methylation, gained through *cis*-antisense transcription driven by *RAG-2* transcription. We have shown that Ikaros can bind to *NWC* promoter and outcompete its activator, ZFP-143 transcription factor, from the common binding sites. Within *RAG/NWC* locus Ikaros has two opposing roles in transcription regulation. It activates *RAG* transcription by binding to *cis*-regulatory elements and to *RAG-1* promoter [22] and downregulates the expression of *NWC* gene.

*Cis*-antisense transcripts spanning the CpG islands are known to have a causative role in establishing the DNA methylation but the exact mechanism of their action is not clear. One possibility is that the observed *cis* effects are due to the ability of the antisense RNA to co-transcriptionally interact with the target DNA to form different kinds of RNA/DNA hybrids which may attract DNA methyltransferases guiding them to the target sequences [24]. Another possibility emerging from the analysis of the mechanism silencing *KIR3DL1* promoter is that antisense transcription driven by a proximal promoter results in the formation of dsRNA which is processed into 28 base PIWI-like RNA [25]. The transcriptional shutdown of a single gene in a cluster of other closely related *KIR* genes would suggest that the antisense RNA is processed rapidly and mediates silencing without diffusion or transport away from the promoter. The chromatin modification accompanying the promoter methylation does not spread across the entire locus thus allowing for unaffected expression of other *KIR* genes [25]. Similar mechanism could be responsible for *NWC* promoter silencing as DNA methylation at the *NWC* promoter changes the chromatin structure only locally and does not affect the transcriptional activity of *RAG* genes [1], [11]. Although because of the bidirectional activity of the transcriptional termination cassette we were not able to directly quantify the effect of *RAG-2* transcription termination on the activity of *NWC* promoter, we expect, based on previous experiments with blocking DNA methylation by 5-azacitidine, that the promoter activity is at least partially restored [11]. The incomplete re-expression of *NWC* in 5-azacitidine-treated lymphoid cells can be explained by the repression of *NWC* promoter by Ikaros and/or transcriptional interference caused by collision *RAG-2* and *NWC* transcription processes.

Taking into consideration the widely accepted view that *RAG* genes “infected” the ancestral genome in the form of a transposon(s), the structure of *NWC* and modes of its regulation described in the present paper, we would like to add new elements to our recent proposal [8]. We propose that *NWC* locus was the original site of integration of *RAG* transposon rather than being part of it, which is suggested by the conserved structure of *RAG/NWC* locus (Fig. S1), multi-exon structure of *NWC* and by the fact that its homologues can be found in various invertebrate species (Fig. S2). Although the conserved NWC protein domains do not share homology to any known protein and the function of the protein is still obscure, the presence of *NWC* gene throughout animal kingdom and its mode of regulation suggest that it is a housekeeping gene. We earlier proposed that constitutive expression of *NWC* as well as the bidirectional activity of its promoter, a feature which characterizes many promoters controlling transposon-derived genes, could initially facilitate the integration and survival of *RAG* transposon, while *cis*-regulatory elements controlling lymphocyte specific expression of *RAG* genes were acquired later in evolution. The localization of the *RAG-2* promoter at the 5′ end of the first exon of *NWC* resulted in convergent and overlapping transcription of *NWC* and *RAG-2* genes, which, if occurred simultaneously, could result in downregulation of both genes owing to transcriptional interference. Such mechanism could inhibit *RAG* expression in lymphocytes below the level required for efficient V(D)J recombination. We think that the expression of Ikaros protein at the initial stage of lymphocyte development in hematopoietic stem cells and its binding to *NWC* promoter causes the displacement of the activator protein ZFP-143 and shifts the equilibrium of opposing transcriptional processes in favor of *RAG-2* transcription, which is additionally activated by Ikaros binding to *RAG* regulatory elements. *RAG-2* transcription proceeding through *NWC* promoter causes its methylation and inactivation without engagement of other factors influencing chromatin accessibility thus preventing the spreading of the changes across the entire locus and enabling undisturbed *RAG* expression.

The evolutionarily conserved association of *NWC* with *RAG-1* and *RAG-2* genes raises additional questions concerning the nature of primordial *RAG* transposon. As a result of the discovery of Transib transposons closely resembling the core fragment of *RAG-1* gene, a modified version of the hypothesis has been put forward, suggesting that only *RAG-1* gene was a part of a mobile element which integrated within *RAG-2* locus [26]. The discussion on the origin of *RAG-1/RAG-2* genes has ignored, however, the existence of *NWC* gene, which most probably was the host gene for *RAG* transposon integration. Given that in invertebrate species *NWC* gene is not associated with any gene resembling *RAG-2* and the bidirectional activity of *NWC* promoter is typical for many host genes controlling transposon-related genes, it is reasonable to assume that the genetic element which integrated within *NWC* locus contained both *RAG-1* and *RAG-2* genes. Interestingly, in *Strongylocentrotus purpuratus*, the only organism outside jawed vertebrates phylum in which both *RAG-1* and *RAG-2* genes were found [27], the *RAG* and *NWC* loci are separate. Since it is highly unlikely that *RAG-1*-containing element integrated twice with *RAG-2* locus this again argues for the “*RAG-1* and *RAG-2*” hypothesis and suggests that the transposon independently infected the ancestors of jawed vertebrates and echinoderms. However, this point of view needs to be verified by extensive analysis of *NWC* transcriptional regulation both in vertebrates and invertebrates.

## Materials and Methods

### Expression and purification of recombinant proteins

PCR-amplified Ikaros cDNA was cloned into *NcoI/XhoI* sites of the pET32a vector to obtain expression vector encoding N-terminal His-tagged recombinant Ikaros protein (pET32a-Ik). In order to express and purify the protein, overnight culture of *E. coli* BL-21 cells transformed with pET32a-Ik was diluted 500 times, cultured at 37^O^C until OD_600_ reached 0.5 and then incubated for the next 2 hours at 4^O^C. Then the cells were induced with IPTG (0.25 mM) and cultured overnight at room temperature in the presence of ZnCl_2_ (100 uM). The bacterial pellet was resuspendend in buffer A, sonicated and applied on HisPur Ni-NTA resin equilibrated with buffer A. Binding the recombinant protein was performed for 1 hour at 4^O^C and then the column was washed several times with buffer B and eluted with buffer C. After elution, the recombinant protein was dialized against buffer D.

Buffer A: 10 mM imidazol, 50 mM Tris-HCl, 300 mM NaCl, 10% glicerol, 0.05% Tween 20, pH 8.0, Buffer B: 20 mM imidazol, 50 mM Tris-HCl, 300 mM NaCl, 10% glicerol, 0.05% Tween 20, pH 8.0, Buffer C: 250 mM imidazol, 50 mM Tris-HCl, 300 mM NaCl, 10% glicerol, 0.05% Tween 20, pH 8.0, Buffer D: 20 mM HEPES pH 7.9, 0.2 mM EDTA, 20% glicerol, 100 mM KCl, 1 mM DTT. All buffers were supplemented with freshly added ZnCl_2_ (20 uM), MgCl_2_ (30 uM), CaCl_2_ (50 uM) and PMSF (1 mM).

ZFP-143 recombinant protein was obtained as previously described [8].

### Electromobility shift assays

The probe containing *NWC* promoter was obtained by amplifying DNA fragment encompassing −119/+12 nucleotides relative to *NWC* transcriptional start site using digoxygenin-labelled primer. PCR ligation method was used to obtain probes with mutations in ZFP-143 and/or Ikaros binding sites. The binding reaction was performed for 20 minutes on ice in 20 ul of reaction mix contaning: binding buffer (20 mM HEPES pH 7.9, 0.2 mM EDTA, 20% glycerol, 100 mM KCl, 1 mM DTT, 10 uM ZnCl_2_), 150 ng of purified recombinant protein, 1 ug poly(dI-dC) and 0.035 pmol of the probe. Where mentioned, 10, 50 or 100x -fold molar excess of unlabelled specific (TCAGCTTT**TGGGAA**TGTATTCCCTGTCA) or non-specific (Oct2) (GGCGTTAAAATTCATTAAAATTCAGGCC) competitor oligonucleotides was added. For ZFP-143/Ikaros competition, 150 ng ZFP-143 was used in the presence of increasing concentration of Ikaros protein (Ikaros/ZFP-143 molar ratio: 0.3, 0.5, 1, 1.5, 2, 2.5, 3, 4). The binding reaction was subjected to electrophoresis in 5% poliacrylamide at 4^O^C. The products were transferred on nylon membrane and detected with anti-digoxygenin antibodies according to manufacturer's recommendations.

### Cell culture and sorting

HEK-293T cells were cultured in DMEM medium (Sigma-Aldrich) supplemented with 10% of FBS (Invitrogen). Jurkat cells were cultured in RPMI medium supplemented with 10% FBS. For Ikaros expression, 0.5×10^6^ HEK-293T cells were plated on 10 cm dish and transfected with 2 ug of Ikaros expressing vector using MetafectanePro reagent (Biontex). The vector was constructed by cloning PCR-amplified Ikaros cDNA into pGEMT-Easy vector (Promega) and then subcloning the *EcoRI* fragment into pLVX-IRES-ZsGreen1 vector (Clontech). After 48 hours ZsGreen1^high^ cells were sorted using FACS-Aria instrument and used for downstream experiments (Real-Time RT-PCR and bisulfite sequencing).

### Real-Time RT-PCR

RNA was isolated from cultured cell lines using TRIzol Reagent (Invitrogen) according to the manufacturer's recommendations. Three micrograms of total RNA was digested with RNase-free DNase I (Thermo Scientific) and reversed transcribed with SuperScript III Reverse Transcriptase (Invitrogen) and random hexamer oligonucleotides at 50°C. Real-Time RT-PCR was performed on a DNA Engine Opticon 2 apparatus (Biorad) using Maxima SYBR Green qPCR Master Mix (Thermo Scientific). The thermal-cycling conditions comprised an initial denaturation step at 95°C for 10 min and 40 cycles of three-step PCR, including 15 s of denaturation at 95°C, 30 s of annealing at 55°C, and 30 s of elongation at 72°C. Expression values were normalized to *HPRT*. Standard curves were prepared for each primer pair by serial 5-fold dilutions of the template cDNA allowing determination of reactions efficiencies. One-way ANOVA followed by Tukey-Kramer post-hoc test was used for statistical analysis.

Primer sequences:

*NWC*: GTCTGCCCATATGTCAGGATTG (forward)

*NWC*: CTCTTCATCCATGTCCAAATCTTC (reverse)

*HPRT*: TGACCTTGATTTATTTGCATACC (forward)

*HPRT*: CGAGCAAGACGTTCAGTCCT (reverse)

### Dual Luciferase Reporter assay

pGL3-Basic based reporter vectors containing *NWC* promoter were obtained as previously described [8]. PCR ligation method was used to obtain constructs with mutations in ZFP-143 and/or Ikaros binding sites. A total of 2×10^5^ HEK293T cells were transfected with 500 ng of firefly luciferase containing reporter plasmids and 50 ng of renilla luciferase containing plasmid (pRL-TK) using MetafectenePro reagent (Biontex) according to the manufacturer's recommendations. The DLR assay was performed 24 h after the transfection using Dual-Luciferase Reporter Assay System reagents (Promega). In the experiments using Ikaros expression vector, the cells were transfected with 500 ng of Ikaros expression vector (pcDNA3-Ikaros, kind gift from S. Smale), 100 ng of firefly luciferase containing reporter plasmids and 50 ng of pRL-TK plasmid, and cultured for 48 hours. The cells were lysed with 100 µl of passive lysis buffer and 15 µl of cell lysates were taken for each analysis. The data is presented as a ratio of firefly (FLU) to renilla (RLU) luciferase activity. One-way ANOVA followed by Tukey-Kramer post-hoc test was used for statistical analysis.

### Bisulfite sequencing

Two micrograms of genomic DNA were treated with HCl (0.1 N) for 2 minutes at room temperature and denatured with NaOH (0.3 M) for 20 minutes at 37°C. The DNA was then treated with sodium bisulfite (35.5%, pH 5.0) in the presence of hydroquinone (0.5 mM) for 5 h at 55°C. The converted DNA was then bound and washed on the Genomic DNA Extraction column (Genoplast) and desulfonated by adding 0.15 NaOH in 90% EtOH on the column and incubating for 10 minutes at room temperature. DNA was washed and eluted from the column. Nested PCR amplification (2×30 cycles) was performed using primer pairs corresponding to the upper strand of the transgenic locus: outer 5′-TTTAAGGAGTTGGGATATGTTTTAGTTA (forward) 5′-ACTCCAACAAAAACAATTATACTTCC (reverse) and inner 5′-GGATATGTTTTTTAGGATTTTTGGG (forward), 5′- AATCACCTATTCAAAAATCCCCAAAA (reverse) or human locus: outer 5′-TCCTCCTAATACTCTTACCTTCCAA (forward) 5′- GGTGGTGTAGATGAATTTTAGGGTTA (reverse) and inner 5′- TCCTAATACTCTTACCTTCCAACACC (forward), 5′- GATTAGGATGGGTATTATTT (reverse)

PCR product was directly cloned into pGEMT-Easy vector (Promega) and at least 20 individual clones were sequenced.

In order to test if PCR amplification of bisulfite-treated DNA did not produce artifacts by selective enrichment of unmethylated or methylated DNA fragments, cubic polynomial regression correction method was used [28]. A calibration experiment was performed based on amplified transgenic locus. A fully methylated PCR fragment was obtained using SssI methylase. Unmethylated and methylated DNA fragments were mixed to obtain DNA of known (0%, 25%, 50%, 75%, 100%) level of methylation and subjected to bisulfite treatment, amplification and cloning as described above.

### Generation of transgenic reporter mouse strains

All procedures using animals were reviewed and approved by First Local Ethical Commission for Animal Experimentation in Wroclaw held in the Institute of Immunology and Experimental Therapy (permit number 13/2009). The mice were sacrificed under sodium thiopental anesthesia.

For the construction of the reporter *RAG-2/NWC* mice strains, a bacterial artificial chromosome encompassing the entire murine RAG/*NWC* locus and expressing *GFP* under the control of *RAG-2* promoter was used (BAC-HG, kind gift from M. Jankovic and M. Nussenzweig, [18]). This BAC was further modified to express *NWC/YFP* fusion protein. The modification was performed as follows: the Neo/Kan resistance cassette encoded by pEGFP-N1 vector (Clontech) was amplified with primers containing *frt* overhangs and cloned together with *YFP* gene in pGEMT-easy vector. The YFP/NeoKan cassette was then amplified with primers harboring 50 nt homology arms corresponding to the region flanking both sides of the STOP codon of *NWC* gene. The amplification product was transformed into bacteria containing BAC-HG and pRed/ET plasmid. The recombination was performed exactly according to the recommendations of the manufacturer of the Red/ET system (Gene Bridges). Obtained clones were verified by PCR, Southern Blot and sequencing to have correct (uninterrupted by the STOP codon), in-frame fusion of *NWC* and *YFP* genes. The construct (BAC-RG/NY) was then transferred to *E.coli* SW105 and the Kan/Neo^r^ cassette was removed by flipase whose expression was induced by arabinose, according to the protocol [29]. BAC-RG/NY was further modified by inserting a transcriptional termination cassette immediately downstream the first exon of *RAG-2* gene to construct BAC-RGterm/NY. The termination cassette consisted of two SV40-polyA sequences linked with twelve *lacO* operators (a kind gift of M.Krangel, [23]). The recombination and selection steps were performed exactly according to the published protocol [29]. Briefly, BAC-RG/NY transformed into *E. coli* SW105 strain was first modified by recombination by inserting the galK cassette flanked by 50 nt homology arms corresponding to the targeted region of *RAG-2* gene and the cells were selected on minimal medium containing galactose as the sole source of carbon. Then the termination cassette flanked by the same homology arms was transformed to the cells and the cells were grown on medium containing glycerol and 2-deoxy-galactose (DOG) in order to select clones in which the termination cassette had replaced the galK cassette. Both constructs (BAC-RG/NY and BAC-RGterm/NY) were linearized with *BseHII*, purified by field inversion gel electrophoresis and used to generate transgenic C57BL/6 mice strains (Karolinska Center for Transgene Technologies, Karolinska Institutet, Stockholm). The transgenic founder mice were then bred in our animal facility and transgenic offspring was used for further experiments. Thymocytes (CD4+CD8+), splenic T-cells (CD4+8-/CD4-8+), splenic B-cells (CD19+), bone marrow preB small cells (small CD25+CD19+) were isolated from 6-week-old mice by staining with anti CD4-PE/anti CD8-APC, CD19-PE, CD25-APC/CD19 PE antibodies (Becton Dickinson), respectively. The cells were sorted with FACS-Aria instrument. Non-lymphoid tissues (brain and liver) were used directly to isolate DNA and its subsequent methylation analysis.

## Supporting Information

Figure S1 **Structure of the** ***RAG/NWC locus*** **in vertebrate species.**
(TIF)

Figure S2 **Multiple sequence alignment of vertebrate full-length NWC proteins and C-terminal portions of vertebrate and invertebrate NWC proteins.**
(TIF)
