# Supplementary figures and images for: Ikaros and RAG-2-Mediated Antisense Transcription Are Responsible for Lymphocyte-Specific Inactivation of NWC Promoter
Source: PLoS One. 2014 Sep 8;9(9):e106927. doi: 10.1371/journal.pone.0106927 (PMC4157847; doi:10.1371/journal.pone.0106927)

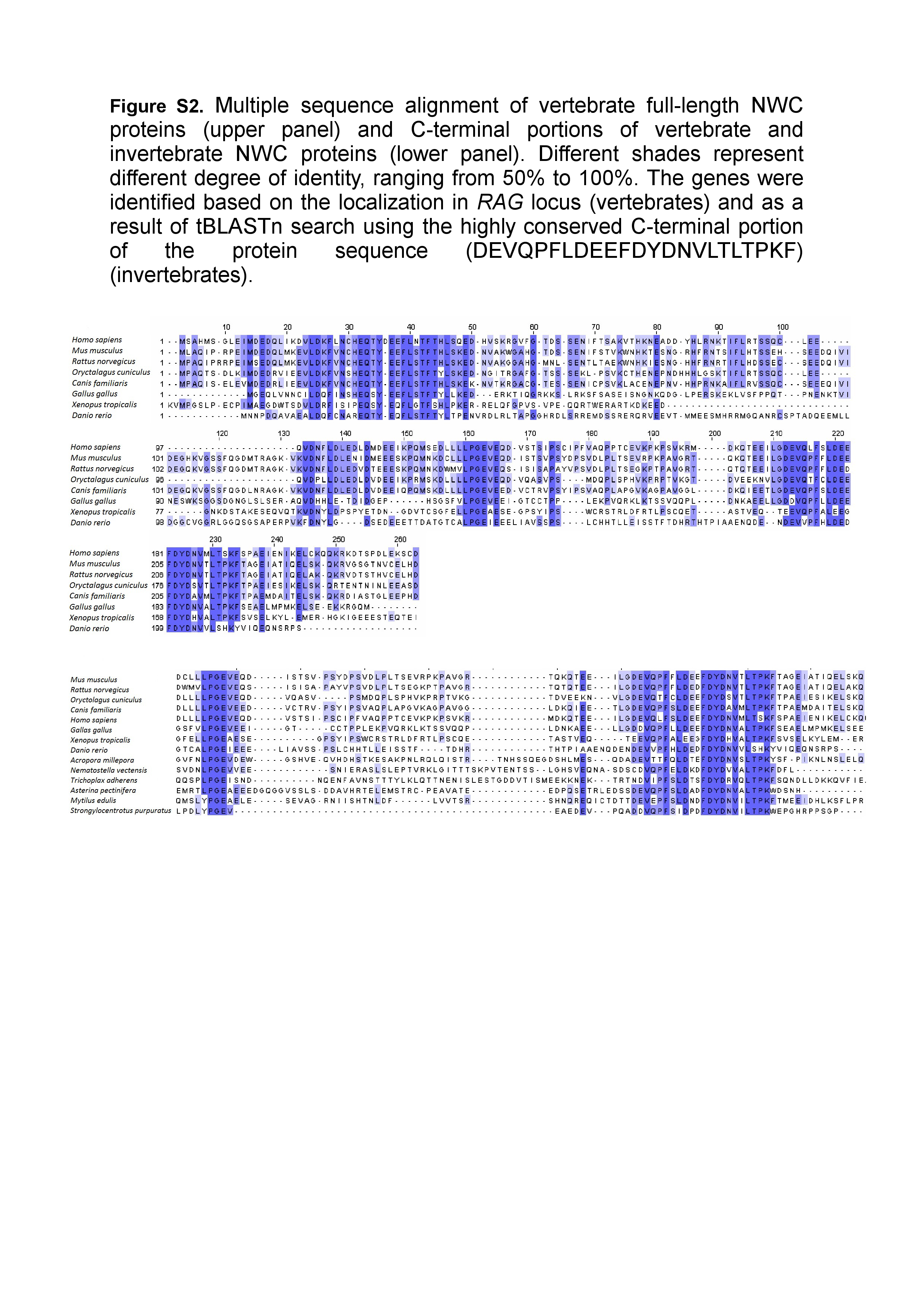

Supplement: Figure S2 — Multiple sequence alignment of vertebrate full-length NWC proteins and C-terminal portions of vertebrate and invertebrate NWC proteins. (TIF) [file pone.0106927.s002.tif]
